# Discriminative Performance of Routine Inflammation- and Nutrition-Based Indices for Histopathologically Confirmed Gastric Malignancy: A Retrospective Case–Control Study

**DOI:** 10.3390/life16091547

**Published:** 2026-09-16

**Authors:** Muammer Bilici, Dilek Ayvaz, Didem Güney, Ünzile Işıl Yüce, Selim Aydemir

**Affiliations:** 1 Department of Internal Medicine, Faculty of Medicine, Zonguldak Bulent Ecevit University, Zonguldak 67100, Turkey; 2 Department of Gastroenterology, Faculty of Medicine, Zonguldak Bulent Ecevit University, Zonguldak 67100, Turkey

**Keywords:** gastric cancer, systemic inflammation, nutritional indices, neutrophil-to-lymphocyte ratio, diagnostic discrimination, internal validation

## Abstract

**Background/Objectives:** This study aimed to evaluate the discriminative performance of routine systemic inflammation- and nutrition-based indices for distinguishing histopathologically confirmed gastric malignancy from non-malignant endoscopic findings. **Methods:** This retrospective case–control study included 211 patients who underwent upper gastrointestinal endoscopy with histopathological evaluation: 139 non-malignant endoscopic controls (129 benign/non-neoplastic and 10 premalignant findings) and 72 gastric malignancy cases. Premalignant lesions were not considered malignant outcomes. Discrimination was evaluated using univariable ROC analyses and multivariable logistic models. The fixed predictor set was assessed using 10-fold out-of-fold validation and 500-resample bootstrap optimism correction; a separate fully nested selection-inclusive audit was used to quantify uncertainty arising from variable selection. **Results:** NLR (AUC = 0.792), INR (AUC = 0.791), and SIRI (AUC = 0.783) showed the highest univariable discrimination. Among the variables retained in Model 1, higher ESR and RDW, lower MPV and HDL, higher INR values, and male sex showed statistically significant multivariable associations. Model 1 had an apparent AUC of 0.928 (95% CI, 0.890–0.959), a fixed-set 10-fold out-of-fold AUC of 0.890, and a bootstrap optimism-corrected AUC of 0.901. A fully nested selection-inclusive audit yielded an out-of-fold AUC of 0.873 and a bootstrap-corrected AUC of 0.869. **Conclusions:** Routine inflammation- and nutrition-related laboratory measures discriminated gastric malignancy from non-malignant endoscopic findings in this endoscopy-selected cohort. The model remains exploratory and requires prospective external validation. These data do not establish screening performance or diagnostic accuracy for early or asymptomatic gastric cancer.

## 1. Introduction

Gastric cancer remains one of the leading causes of cancer-related mortality worldwide. Its prognosis is largely unfavorable, primarily due to late-stage diagnosis in a substantial proportion of patients [1]. While survival outcomes are significantly improved when the disease is detected at an early stage, patients diagnosed at advanced stages face limited therapeutic options and high mortality rates [2]. Therefore, early detection of gastric cancer plays a critical role in improving clinical outcomes.

Upper gastrointestinal endoscopy with histopathological evaluation remains the diagnostic reference standard for gastric cancer [3]. Routine laboratory measures cannot replace this standard; however, their evaluation in an endoscopy-referred population may provide complementary information on group-level discrimination between malignant and non-malignant findings [4].

In recent years, the role of systemic inflammation in cancer development and progression has been increasingly recognized. The interaction between the tumor microenvironment and systemic inflammatory response plays a pivotal role in tumor proliferation, angiogenesis, and metastasis [5]. In this context, hematological indices such as the neutrophil-to-lymphocyte ratio (NLR), systemic immune-inflammation index (SII), and systemic inflammation response index (SIRI) have been investigated as diagnostic and prognostic biomarkers across various malignancies [6,7].

In addition to inflammation, nutritional status constitutes another critical component of cancer biology. Malnutrition has been associated with impaired immune response, reduced treatment tolerance, and poorer clinical outcomes [8]). Accordingly, nutrition-based parameters such as the Controlling Nutritional Status (CONUT) score, Hemoglobin–Albumin–Lymphocyte–Platelet (HALP) index, and Prognostic Nutritional Index (PNI) have gained increasing attention in oncological research [9].

However, the existing literature predominantly focuses on individual inflammatory or nutritional biomarkers, whereas studies evaluating these parameters within an integrated biological framework remain limited. Moreover, the frequent use of healthy individuals as control groups may not adequately reflect real-world clinical settings, thereby limiting the external validity and practical applicability of reported diagnostic performances [10].

In this study, we evaluated the discriminative performance of systemic immune–inflammatory and nutrition-based indices for distinguishing histopathologically confirmed gastric malignancy from non-malignant endoscopic findings in a clinically relevant endoscopy-selected population. We additionally examined whether composite indices or multivariable combinations improved discrimination beyond individual markers. The study was not designed to assess population screening or early/asymptomatic gastric cancer detection.

## 2. Materials and Methods

### 2.1. Study Design and Patient Population

This retrospective case–control study assessed 262 adults who underwent upper gastrointestinal endoscopy with histopathological evaluation at the Department of Gastroenterology during 2022–2024. The primary endpoint was histopathologically confirmed invasive gastric malignancy. Benign/non-neoplastic and premalignant findings were classified as non-malignant endoscopic controls; premalignant lesions were not classified as malignant outcomes. The final analytic cohort comprised 211 patients (139 non-malignant controls and 72 malignant cases).

The available study documentation did not establish whether enrollment was consecutive or provide a standardized distribution of endoscopy indications. Accordingly, the sample is described as an endoscopy-selected retrospective case–control cohort, without a claim of consecutive recruitment or representativeness of all endoscopy referrals.

Of 262 assessed patients, 51 were excluded: active systemic infection (*n* = 15), hematological malignancy (*n* = 5), recent major surgery or blood transfusion (*n* = 6), advanced hepatic or renal failure (*n* = 7), chronic immunosuppressive or corticosteroid therapy (*n* = 2), and missing laboratory data required for analysis (*n* = 16). The reported exclusions sum to 51 and leave 211 participants. Participant flow is shown in Supplementary Figure S1; an item-by-item TRIPOD + AI reporting checklist identifies both reported information and remaining documentation limitations (Supplementary Section S5) [11].

All included participants underwent endoscopic and histopathological evaluation. Inclusion required age ≥18 years and availability of endoscopic, histopathological, and laboratory data needed for analysis. The final analytic dataset contained no missing entries in its 55 recorded fields, including the 45 candidate predictors examined in the supplementary selection audit. Each candidate was available for all 139 controls and 72 cases; no imputation was performed. Variable-level missingness in the included cohort is reported in Supplementary Table S1. Variable-specific missingness and outcome-group information for the 16 patients excluded for incomplete laboratory data were not available for analysis; therefore, selection associated with missingness could not be assessed.

Propensity score matching was not used because the objective was diagnostic discrimination rather than estimation of a causal treatment or exposure effect. Age and sex were retained in the multivariable models. Matching would change the observed case mix and could discard eligible observations without establishing representativeness or resolving unmeasured confounding. The reported associations are not interpreted causally [12].

### 2.2. Data Collection and Definitions

Demographic, clinical, and laboratory data were retrospectively retrieved from hospital records. Laboratory measurements were obtained within a maximum of three days before endoscopy and before cancer-directed treatment; the interval was verified for each patient from medical records. Acute gastrointestinal bleeding was assessed using the presenting history, clinical evaluation, acute decreases in hemoglobin, and endoscopic findings. Recent localized infection was assessed using history, physical examination, and clinical and laboratory findings; the investigators reported exclusion of patients with suspected active or recent localized infection. However, a separate count for localized infection, its overlap with the reported systemic-infection exclusions, and patient-level bleeding indicators were not available in the analytical materials. The flow diagram therefore reproduces the documented exclusion totals without adding an unverified exclusion category. These assessments do not establish that every participant with bleeding was excluded.

Serum CRP was measured by an immunoturbidimetric assay on a Roche Cobas c501/c502 platform, expressed in mg/L, with an institutional reference interval of 0–5 mg/L. Contemporaneous institution-specific reference intervals for the other assays were not documented in the materials available for this revision; laboratory values are consequently interpreted as comparative measurements rather than classified as clinically abnormal on the basis of study-derived thresholds.

Medication exposures (including anticoagulants, statins, and NSAIDs) were not available as systematically recorded variables in the analytical database and could not be adjusted for reliably; this was addressed as a source of residual confounding.

### 2.3. Inflammation and Nutritional Indices

Indices reflecting systemic inflammation and nutritional status were calculated from routine laboratory parameters. Inflammatory indices included the neutrophil-to-lymphocyte ratio (NLR), systemic immune-inflammation index (SII), systemic inflammation response index (SIRI), and pan-immune-inflammation value (PIV). Nutritional status was assessed using the Controlling Nutritional Status (CONUT) score and the Hemoglobin–Albumin–Lymphocyte–Platelet (HALP) index.

To ensure comparability across measurement scales, the component variables used to construct the composite scores were standardized from their observed raw values as Z = (X − μ)/s, where μ and s denote the sample mean and sample standard deviation. The CNIS reconstruction used these raw-value Z-scores rather than log-transformed components. The inflammation Z-score was calculated as (Z_PIV + Z_CAR + Z_SII + Z_NLR)/4.

To represent nutritional burden, the following formula was applied:

Nutrition Z-score = (Z_CONUT − Z_HALP − Z_albumin)/3

To evaluate the combined effects of inflammation and malnutrition, composite indices were defined. The Composite Nutrition–Inflammation Score (CNIS) was calculated as:

CNIS = (Z_PIV + Z_CAR + Z_SII + Z_NLR + Z_CONUT) − (Z_HALP + Z_albumin)

As a more parsimonious alternative, the Inflammation–Nutrition Index (IINI) was defined as:

IINI = (Z_PIV + Z_CAR + Z_CONUT) − Z_HALP

For Model 1, HALP was dichotomized as high (>median) or low (≤median). For Model 2, CNIS groups were low (≤25th percentile), intermediate (>25th and <75th percentile), and high (≥75th percentile, reference). Model 2 replaced the CONUT and HALP terms of Model 1 with two CNIS indicators; it was not an extension of Model 1 containing an additional CNIS term. Continuous model predictors were standardized using training-sample means and population-standard-deviation scaling (ddof = 0), whereas composite-score component Z-scores used sample standard deviations (ddof = 1). All relevant scaling constants and category cut-points were re-estimated within each resampling training set. Full-cohort constants and model equations are given in Supplementary Section S3.

### 2.4. Statistical Analysis

All analyses were performed using Python (version 3.12.4). Data processing and numerical computations used pandas and numpy; statistical tests used scipy.stats and statsmodels; and penalized regression, ROC analysis, and resampling used scikit-learn. All tests were two-sided, and *p* < 0.05 was considered statistically significant.

Baseline and laboratory characteristics were compared between outcome groups, with descriptive effect sizes accompanying hypothesis tests. These group comparisons are exploratory and do not establish causal effects. The previously reported primary predictor set was retained to avoid an additional post hoc selection step; its reproducibility and selection-related uncertainty were addressed separately as described below.

### 2.5. Diagnostic Performance Analysis

The univariable discriminative ability of each marker was evaluated using ROC analysis. Confidence intervals for multivariable apparent and out-of-fold AUCs were estimated from 5000 class-stratified bootstrap samples of the corresponding predictions; these intervals do not incorporate all variability from repeated model development. Univariable AUC estimates and standard errors were retained. Youden cut-offs were derived and evaluated in the same dataset and are exploratory, study-specific thresholds, not validated clinical decision limits. Classification used the unrounded values; precision-sensitive thresholds are supplied in Supplementary Table S4. Corresponding odds ratios are unadjusted. PPV and NPV reflect the malignancy proportion in this case–control sample and should not be transported directly to a target clinical population. Retaining continuous measurements for ROC analysis avoids requiring externally imposed dichotomies [13].

### 2.6. Model Development

The primary model comprised 12 parameters retained in the original analysis, which was reported as LASSO-based: age, ESR, hematocrit, RDW, MPV, HDL cholesterol, ALT, ALP, INR, CONUT score, sex, and dichotomized HALP. The archived materials documented the retained set but did not contain the original code, complete candidate pool, or lambda-tuning trace; those historical details were not reconstructed by assumption. The primary models were refitted using these fixed sets. Only coefficients with *p* < 0.05 are described as statistically significant multivariable associations. Wald statistics after data-driven selection are descriptive and do not account for selection uncertainty; variables were not deleted post hoc solely on the basis of these *p*-values.

A separate selection-inclusive methodological sensitivity analysis considered 45 available baseline candidate variables: age, sex, Helicobacter pylori status, number of comorbidities, history of extra-gastric malignancy, PIV, CAR, CONUT, HALP, SII, SIRI, ESR, CEA, NLR, PLR, WBC, hemoglobin, hematocrit, platelet count, MCV, MCH, MCHC, RDW, RDW-SD, MPV, neutrophil, lymphocyte, monocyte, eosinophil and basophil counts, HDL, LDL, triglycerides, total cholesterol, CRP, ALT, AST, GGT, ALP, INR, urea, creatinine, total protein, albumin, and calcium. Post-diagnostic stage, treatment, follow-up, and mortality variables were excluded; AISI was excluded because it was numerically identical to PIV. The timing of ascertainment of Helicobacter pylori status was not established, so this supplementary audit is not presented as validation of a wholly pre-endoscopic clinical model.

In this audit, all candidate features were standardized within the training data. L1-penalized logistic regression was tuned over 12 logarithmically spaced inverse-penalty C values from 10^−2.5^ to 10^0.8^. Inner stratified five-fold cross-validation used ROC AUC and selected the smallest C within one standard error of the best mean AUC. Nonzero selected coefficients were then refitted in an unpenalized logistic model. The complete preprocessing, tuning, selection, and refitting procedure was repeated within each outer ten-fold training set and each of 500 bootstrap samples [14]. This explicitly specified supplementary procedure evaluates selection uncertainty but is not a reconstruction of the unavailable historical LASSO pipeline.

For the primary model, fixed-set internal validation refitted preprocessing and model coefficients within each training or bootstrap sample but kept the previously selected predictor set fixed. This analysis is explicitly reported as conditional on the selected predictor set and does not include selection uncertainty. Apparent and out-of-fold AUCs, Brier score, calibration intercept, and calibration slope were calculated. Harrell’s bootstrap optimism correction used 500 resamples.

Sensitivity analyses (i) refitted both models after excluding the 10 premalignant controls and (ii) restricted the malignant group to the 52 records explicitly categorized as gastric adenocarcinoma, compared with 139 non-malignant controls. The four records carrying an unverified early gastric cancer (EGC) label and the 16 other malignancies were excluded from the adenocarcinoma-restricted analysis because lesion-level histology could not be established from the analytic categories. Exploratory TNM I–II and III–IV discrimination estimates were calculated from the relevant subsets of full-cohort out-of-fold predictions, not from separately developed stage-specific models. These comparisons do not estimate EGC performance.

## 3. Results

### 3.1. Patient Characteristics

The 211 included records comprised 129 benign/non-neoplastic findings (61.1%), 10 premalignant findings (4.7%), four gastric malignancy records carrying an unverified EGC label (1.9%), 52 gastric adenocarcinomas (24.6%), and 16 other malignancies (7.6%). The primary comparison retained the original binary outcome: 139 non-malignant controls (categories 1–2) and 72 malignancies (categories 3–5). These were mutually exclusive database categories; the four category-3 records were not additionally counted among the 52 category-4 records.

The available analytic categories did not resolve the lesion-level diagnoses within the benign/non-neoplastic, premalignant, or other-malignancy groups. The four EGC labels could not be independently confirmed because the required depth-of-invasion information was not available for this revision. EGC is defined by invasion limited to the mucosa or submucosa, irrespective of nodal involvement, and is not equivalent to TNM stage I or I–II. Higher recorded TNM stages alone therefore do not demonstrate a coding error [3,15]. No unverified histological subtype or EGC-specific diagnostic estimate was assigned.

Patients with gastric malignancy were significantly older than non-malignant endoscopic controls (66.9 ± 11.6 vs. 57.4 ± 12.8 years, *p* < 0.001, Cohen’s d = 0.80), indicating a large effect size. Sex distribution also differed significantly between groups, with a higher proportion of males among patients with gastric malignancy (76.4% vs. 46.8%, *p* < 0.001). Helicobacter pylori status, comorbidity burden, comorbidity presence, history of extra-gastric malignancy, and number of comorbidities did not differ significantly between groups.

Post-diagnostic variables were not included in baseline comparisons. Among the 72 malignant cases, TNM stage was I in 5 (6.9%), II in 18 (25.0%), III in 28 (38.9%), and IV in 21 (29.2%) patients; thus, 23 (31.9%) had TNM stage I–II disease and 49 (68.1%) had TNM stage III–IV disease. These TNM groupings are not synonymous with the pathological definition of early gastric cancer.

In exploratory stage-stratified descriptions, median NLR values were 1.54, 3.10, and 3.62 in controls, TNM I–II cases, and TNM III–IV cases, respectively; corresponding SIRI medians were 0.88, 1.69, and 2.07, HDL medians were 54, 40, and 38 mg/dL, and HALP medians were 38.88, 36.10, and 19.38. Using subsets of the full-cohort conditional out-of-fold predictions, AUCs were 0.873 (95% CI, 0.787–0.944) for TNM I–II versus controls and 0.898 (95% CI, 0.846–0.942) for TNM III–IV versus controls. These are descriptive subgroup estimates, not a test establishing equal performance across stages; they cannot establish early-cancer detection.

After exclusion of the 10 premalignant controls and refitting of the models (*n* = 201), apparent AUCs remained similar for Model 1 (0.925; 95% CI, 0.887–0.958) and Model 2 (0.924; 95% CI, 0.884–0.956), (Table 1).

### 3.2. Laboratory Parameters

Comparative analysis of laboratory parameters demonstrated a distinct hematological, inflammatory, and nutritional profile in patients with gastric malignancy.

Markers of anemia were significantly more pronounced in the malignant group, with lower hemoglobin and hematocrit levels (both *p* < 0.001). Evaluation of erythrocyte indices revealed reduced mean corpuscular volume (MCV) and mean corpuscular hemoglobin (MCH), accompanied by a significant increase in red cell distribution width (RDW) (all *p* < 0.001), indicating greater heterogeneity in erythrocyte morphology.

Leukocyte subset analysis showed higher neutrophil counts and lower lymphocyte counts in the malignant group (both *p* < 0.001). CRP and INR values were also higher in the malignant group (both *p* < 0.001); these findings are observational and do not establish tumor-specific causation.

Nutritional assessment demonstrated significantly lower total protein and albumin levels in the malignant group (*p* < 0.001), reflecting a compromised nutritional and metabolic status. Within the lipid profile, high-density lipoprotein (HDL) cholesterol levels were significantly reduced (*p* < 0.001), whereas no statistically significant differences were observed for low-density lipoprotein (LDL) cholesterol or triglyceride levels between the groups (Table 2).

**Table 2:** Comparison of laboratory, inflammatory, and biochemical parameters between gastric malignancy cases and non-malignant endoscopic controls, with standardized effect sizes.

Variable	Total (*n* = 211)	Non-Malignant Controls (*n* = 139)	Gastric Malignancy (*n* = 72)	Effect Size (d Equivalent)	*p*
WBC, ×10^9^/L, median (Q1/Q3)	7.94 (6.22/9.39)	8.10 (6.45/9.63)	7.30 (6.05/9.12)		0.120 ^a^
Hemoglobin, g/dL, mean (SD)	12.05 (2.15)	12.67 (1.89)	10.86 (2.12)	0.9	<0.001 †
Hematocrit, %, mean (SD)	37.16 (6.44)	39.01 (5.66)	33.59 (6.37)	0.9	<0.001 †
Platelet count, ×10^9^/L, median (Q1/Q3)	254 (203.5/316)	251 (215/311.5)	255.5 (203/329.5)		0.992 ^a^
MCV, fL, median (Q1/Q3)	84.9 (80.2/89.5)	87.20 (81.75/89.8)	80.85 (75.92/85.62)	0.9	<0.001 ^a^
MCH, pg, median (Q1/Q3)	28.3 (26.1/29.8)	28.50 (26.75/30.05)	26.5 (24.40/28.7)	0.8	<0.001 ^a^
MCHC, g/dL, median (Q1/Q3)	32.9 (32.0/33.8)	32.90 (32.15/33.75)	32.55 (31.8/33.8)		0.340 ^a^
RDW, %, median (Q1/Q3)	14.9 (13.5/17.0)	14.30 (13.20/16.30)	16.3 (14.5/20.1)	1.0	<0.001 ^a^
RDW-SD, fL, median (Q1/Q3)	44.8 (41.60/50.1)	43.70 (41.6/48.1)	47.9 (42.40/55.6)	0.5	0.008 ^a^
MPV, fL, median (Q1/Q3)	8.90 (8.30/9.80)	9.20 (8.40/10.10)	8.60 (7.88/9.12)	0.7	<0.001 ^a^
Neutrophil count, ×10^9^/L, median (Q1/Q3)	4.30 (2.79/5.65)	3.92 (2.40/5.27)	4.80 (3.80/6.53)	0.7	<0.001 ^a^
Lymphocyte count, ×10^9^/L, median (Q1/Q3)	1.87 (1.33/2.50)	2.02 (1.52/2.73)	1.54 (1.08/1.92)	0.8	<0.001 ^a^
Monocyte count, ×10^9^/L, median (Q1/Q3)	0.59 (0.42/0.70)	0.54 (0.40/0.70)	0.60 (0.50/0.70)	0.3	0.044 ^a^
Eosinophil count, ×10^9^/L, median (Q1/Q3)	0.18 (0.10/0.30)	0.18 (0.09/0.26)	0.11 (0.10/0.30)		0.840 ^a^
Basophil count, ×10^9^/L, median (Q1/Q3)	0.03 (0.01/0.10)	0.03 (0.01/0.06)	0.08 (0.01/0.10)		0.208 ^a^
HDL cholesterol, mg/dL, median (Q1/Q3)	46 (36.50/59)	54 (41/67)	38.5 (30.7/44.3)	1.2	<0.001 ^a^
LDL cholesterol, mg/dL, median (Q1/Q3)	82 (55/112)	73 (48/112.5)	89.5 (61.7/111)		0.116 ^a^
Triglycerides, mg/dL, median (Q1/Q3)	119 (87/157)	115 (85/158)	124 (90.7/150.7)		0.424 ^a^
Total cholesterol, mg/dL, median (Q1/Q3)	165 (131/196)	173 (136.5/208.5)	154.5 (123/179)	0.5	<0.001 ^a^
CRP, mg/L, median (Q1/Q3)	7.53 (3.58/17.55)	5.70 (2.94/13.93)	14.10 (6.50/29.51)	0.9	<0.001 ^a^
ALT, U/L, median (Q1/Q3)	14 (10/22)	15 (10/23)	13 (9/19.25)	0.4	0.032 ^a^
AST, U/L, median (Q1/Q3)	20 (15/26)	21 (16/25)	18 (13/26.25)		0.292 ^a^
GGT, U/L, median (Q1/Q3)	23 (15/35)	24 (16/34.50)	19 (13/40.50)		0.300 ^a^
ALP, U/L, median (Q1/Q3)	76 (60/96)	72 (58/93.50)	84 (66.25/106.5)	0.4	0.012 ^a^
INR, dimensionless, median (Q1/Q3)	0.99 (0.93/1.09)	0.96 (0.90/1.02)	1.08 (1.01/1.17)	1.4	<0.001 ^a^
Urea, mg/dL, median (Q1/Q3)	33 (28/45)	33 (28/44.50)	34 (27/46.75)		0.548 ^a^
Creatinine, mg/dL, median (Q1/Q3)	0.80 (0.70/1)	0.80 (0.70/0.96)	0.90 (0.70/1.02)		0.324 ^a^
Total protein, g/dL, median (Q1/Q3)	6.70 (6.10/7.10)	6.80 (6.30/7.10)	6.40 (5.90/6.93)	0.6	<0.001 ^a^
Albumin, g/dL, median (Q1/Q3)	4 (3.40/4.30)	4.10 (3.50/4.35)	3.70 (3.27/4.10)	0.6	<0.001 ^a^
Calcium, mg/dL, median (Q1/Q3)	9.30 (8.90/9.70)	9.40 (9.10/9.80)	9.20 (8.80/9.62)		0.092 ^a^

^a^ Mann–Whitney U test (Monte Carlo); † independent-samples *t* test (bootstrap). SD: standard deviation; Q1: first quartile; Q3: third quartile. CRP: serum immunoturbidimetric assay, Roche Cobas c501/c502; institutional reference interval 0–5 mg/L. Effect sizes were initially computed according to variable type: Cohen’s d for continuous variables, rank-biserial correlation for Mann–Whitney U comparisons. For consistency and comparability across variables, these effect sizes were subsequently transformed to an equivalent Cohen’s d metric using standard conversion formulas (approximations for categorical variables). Non-malignant endoscopic controls included benign and premalignant histopathological findings without invasive malignancy.

### 3.3. Inflammation and Nutritional Index Profiles

Comparative analyses demonstrated a consistent and statistically significant deterioration across the majority of immune–inflammatory and nutrition-based systemic indices in the malignant group.

Inflammatory indices, including the pan-immune-inflammation value (PIV), C-reactive protein-to-albumin ratio (CAR), systemic immune-inflammation index (SII), systemic inflammation response index (SIRI), and neutrophil-to-lymphocyte ratio (NLR), were all significantly elevated in patients with malignancy (all *p* < 0.001). In contrast, the Hemoglobin–Albumin–Lymphocyte–Platelet (HALP) index was significantly lower in the malignant group (*p* < 0.001), indicating an impaired nutritional and immunological status.

Evaluation of composite indices further supported these findings. Both the Composite Nutrition–Inflammation Score (CNIS) and the Inflammation–Nutrition Index (IINI) were significantly higher in the malignant group (*p* < 0.001). In parallel, the Nutrition Z-score was also significantly increased in malignant patients, reflecting a greater burden of malnutrition as defined by the applied standardization framework.

The high-risk CNIS category was more frequent in the malignant group (41.7% vs. 16.5%, *p* < 0.001). In contrast, the established categorical CONUT classification was not significantly different between groups (*p* = 0.075), despite a difference in the continuous CONUT score. This divergence may reflect information loss after categorization and the small severe-CONUT subgroup (Table 3).

### 3.4. Diagnostic Performance (ROC Analysis)

Univariable ROC analysis showed moderate discrimination for NLR (AUC = 0.792), INR (AUC = 0.791), and SIRI (AUC = 0.783). HDL cholesterol (AUC = 0.764), SII (AUC = 0.754), PIV (AUC = 0.740), hemoglobin (AUC = 0.737), hematocrit (AUC = 0.737), ESR (AUC = 0.728), RDW (AUC = 0.722), CNIS (AUC = 0.714), and IINI (AUC = 0.714) provided lower discrimination. Markers with AUC < 0.70 were interpreted as having limited rather than clinically meaningful discrimination.

After exclusion of premalignant lesions, the principal single-marker AUCs were materially unchanged: NLR = 0.792, INR = 0.785, SIRI = 0.780, HDL cholesterol = 0.759, and SII = 0.759.

**Figure 1:**
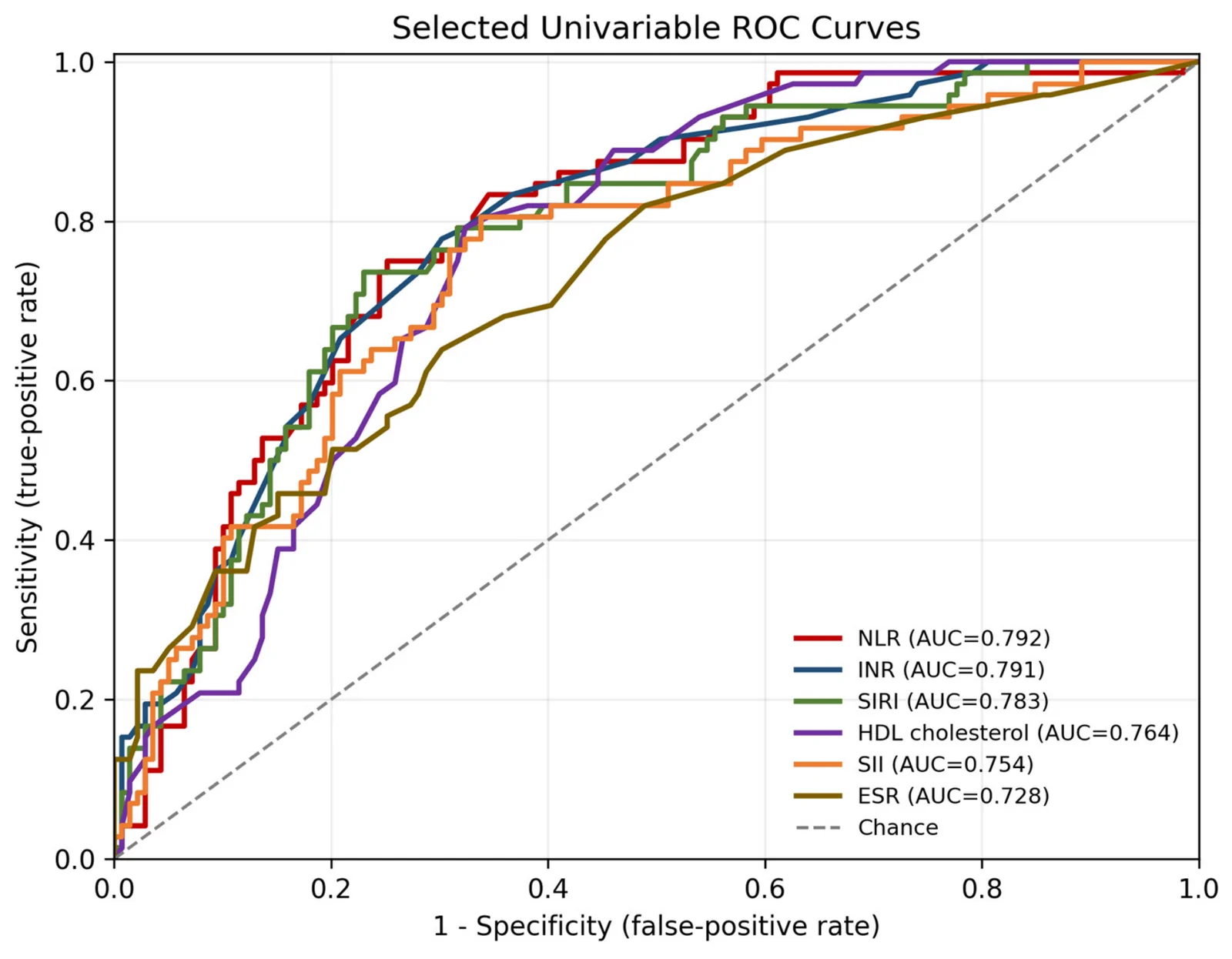
Univariable ROC curves of selected laboratory-derived markers for gastric malignancy discrimination.

All univariable cut-offs and associated classification measures were derived and evaluated in the same sample and should therefore be regarded as exploratory and potentially optimistic. Complete univariable ROC results are provided in Table 4, and selected univariable ROC curves are shown in Figure 1.

**Table 4:** Univariable receiver operating characteristic analysis of demographic, laboratory, inflammatory, and nutritional parameters for discriminating gastric malignancy cases from non-malignant endoscopic controls.

Marker	Cut-off	Sens. (%)	Spec. (%)	PPV (%)	NPV (%)	AUC ± SE	Unadjusted OR (95% CI)	Cohen’s d	*p*
Age	≥66.00	62.50	73.38	54.88	79.07	0.713 ± 0.037	4.6 (2.5–8.4)	0.80	<0.001
CAR	≥0.18	75.00	61.15	50.00	82.52	0.704 ± 0.036	4.7 (2.5–8.9)	0.76	<0.001
CONUT Score	≥1.00	91.67	33.81	41.77	88.68	0.652 ± 0.038	5.6 (2.3–13.9)	0.55	<0.001
HALP Index	≤36.63	76.39	56.12	47.41	82.11	0.703 ± 0.037	4.1 (2.2–7.8)	0.75	<0.001
SII	≥507.69	80.56	66.19	55.24	86.79	0.754 ± 0.036	8.1 (4.1–16.0)	0.97	<0.001
SIRI	≥1.44	73.61	76.98	62.35	84.92	0.783 ± 0.032	9.3 (4.8–18.0)	1.11	<0.001
PIV	≥343.79	69.44	69.06	53.76	81.36	0.740 ± 0.036	5.1 (2.7–9.4)	0.91	<0.001
ESR	≥13.00	63.89	69.78	52.27	78.86	0.728 ± 0.036	4.1 (2.2–7.5)	0.86	<0.001
CEA	≥2.47	54.17	87.77	69.64	78.71	0.711 ± 0.039	8.5 (4.3–16.9)	0.79	<0.001
NLR	≥2.35	75.00	74.82	60.67	85.25	0.792 ± 0.031	8.9 (4.6–17.2)	1.15	<0.001
PLR	≥191.30	45.83	81.29	55.93	74.34	0.656 ± 0.039	3.7 (2.0–6.9)	0.57	<0.001
Hemoglobin	≤11.10	56.94	81.29	61.19	78.47	0.737 ± 0.036	5.7 (3.1–10.8)	0.90	<0.001
Hematocrit	≤34.30	56.94	81.29	61.19	78.47	0.737 ± 0.036	5.7 (3.1–10.8)	0.90	<0.001
MCV	≤84.60	73.61	64.03	51.46	82.41	0.714 ± 0.037	5.0 (2.6–9.3)	0.80	<0.001
MCH	≤27.80	65.28	64.03	48.45	78.07	0.680 ± 0.039	3.3 (1.8–6.1)	0.66	<0.001
RDW	≥15.10	69.44	62.59	49.02	79.82	0.722 ± 0.039	3.8 (2.1–7.0)	0.83	<0.001
RDW SD	≥49.00	47.22	79.86	54.84	74.50	0.628 ± 0.045	3.5 (1.9–6.6)	0.46	0.004
MPV	≤9.10	75.00	52.52	45.00	80.22	0.673 ± 0.037	3.3 (1.8–6.2)	0.63	<0.001
Neutrophil count	≥3.20	87.50	38.85	42.57	85.71	0.659 ± 0.037	4.4 (2.0–9.7)	0.58	<0.001
Lymphocyte count	≤1.90	75.00	55.40	46.55	81.05	0.683 ± 0.037	3.7 (2.0–7.0)	0.67	<0.001
Monocyte count	≥0.50	80.56	37.41	40.00	78.79	0.585 ± 0.039	2.5 (1.3–4.9)	0.30	0.028
HDL Cholesterol	≤45.00	79.17	67.63	55.88	86.24	0.764 ± 0.032	7.9 (4.1–15.5)	1.02	<0.001
Total Cholesterol	≤179.00	76.39	46.04	42.31	79.01	0.614 ± 0.041	2.8 (1.5–5.2)	0.41	0.006
CRP	≥7.76	72.22	62.59	50.00	81.31	0.701 ± 0.036	4.3 (2.3–8.1)	0.75	<0.001
ALT	≤14.00	61.11	55.40	41.51	73.33	0.591 ± 0.042	2.0 (1.1–3.5)	0.33	0.030
ALP	≥77.00	62.50	57.55	43.27	74.77	0.605 ± 0.042	2.3 (1.3–4.1)	0.38	0.012
INR	≥1.01	77.78	69.78	57.14	85.84	0.791 ± 0.032	8.1 (4.2–15.7)	1.15	<0.001
Total Protein	≤6.50	63.89	66.91	50.00	78.15	0.652 ± 0.041	3.6 (2.0–6.5)	0.55	<0.001
Albumin	≤3.90	66.67	58.99	45.71	77.36	0.635 ± 0.039	2.9 (1.6–5.2)	0.49	<0.001
Nutrition Z-score	≥−0.29	79.17	53.24	46.72	83.15	0.681 ± 0.037	4.3 (2.2–8.4)	0.67	<0.001
CNIS	≥−0.29	68.06	69.06	53.26	80.67	0.714 ± 0.035	4.8 (2.6–8.8)	0.80	<0.001
IINI	≥−0.56	77.78	58.27	49.12	83.51	0.714 ± 0.035	4.9 (2.6–9.4)	0.80	<0.001

SE: standard error; PPV: positive predictive value; NPV: negative predictive value. Odds ratios are unadjusted. Cut-offs were derived and evaluated in the same dataset using the Youden index and are exploratory study-specific thresholds, not validated clinical decision limits. PPV and NPV reflect the malignancy proportion in this case–control sample. Sens.: sensitivity; Spec.: specificity; OR: odds ratio. Inequalities are inclusive (≥ or ≤). Displayed cut-offs are rounded; classification used unrounded values. Precision-sensitive cut-offs are given in Supplementary Table S4.

### 3.5. Multivariable Modeling

The primary multivariable model contained 12 LASSO-selected parameters. Within this model, higher ESR and RDW, lower MPV and HDL cholesterol, higher INR values, and male sex showed statistically significant multivariable associations (all *p* < 0.05). Age, hematocrit, ALT, ALP, CONUT, and HALP were retained model terms but were not described as statistically significant independent associations.

Model 1 had an apparent AUC of 0.928 (95% CI, 0.890–0.959), AIC of 161.948, and Brier score of 0.102. The unrounded AUC was 0.92755795; Table 5 provides four-decimal precision for model comparisons. At the internally derived Youden threshold of 0.4698, sensitivity was 81.9%, specificity 89.2%, PPV 79.7%, NPV 90.5%, and accuracy 86.7%.

Model 2 replaced the CONUT and HALP terms with CNIS categories. Its apparent AUC was 0.9272 (95% CI, 0.8896–0.9580), with AIC 161.922. At its Youden threshold of 0.5089, sensitivity was 77.8%, specificity 92.1%, PPV 83.6%, NPV 88.9%, and accuracy 87.2%. The paired bootstrap AUC difference (Model 1 minus Model 2) was 0.0004 (95% CI, −0.0053 to 0.0063; *p* = 0.888), showing no discernible discrimination advantage for the CNIS-based alternative. This was not a nested-model test of adding CNIS to Model 1.

Conditional on the previously selected predictor set, Model 1 had a 10-fold out-of-fold AUC of 0.8897 (95% CI, 0.8406–0.9307), Brier score of 0.126, calibration intercept of −0.128, and calibration slope of 0.712. Mean bootstrap optimism was 0.0266, yielding an optimism-corrected AUC of 0.9009. Corresponding Model 2 values were an out-of-fold AUC of 0.8914 (95% CI, 0.8446–0.9322) and a bootstrap-corrected AUC of 0.8996.

The selection-inclusive audit yielded an apparent AUC of 0.9273, a nested ten-fold out-of-fold AUC of 0.8730 (95% CI, 0.8202–0.9202), and a bootstrap optimism-corrected AUC of 0.8694 (mean optimism, 0.0578). The out-of-fold Brier score was 0.144, calibration intercept −0.300, and calibration slope 0.406. The median number of selected predictors across bootstrap samples was 21 (IQR, 17–26), indicating substantial selection instability. These findings demonstrate material overfitting and uncertainty; the audit neither externally validates the model nor verifies its historical selection procedure.

**Figure 2:**
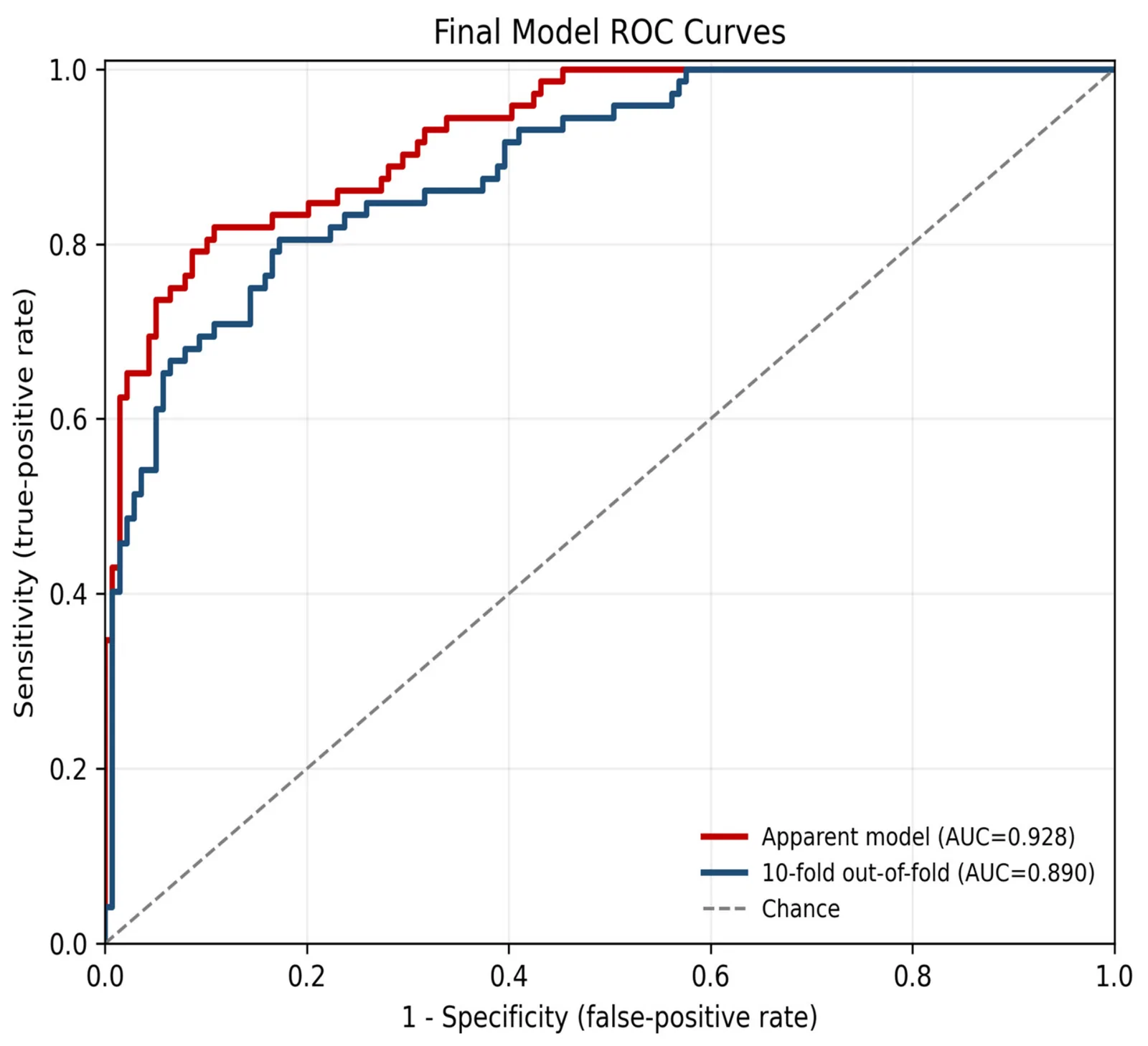
Receiver operating characteristic curves for the final Model 1: apparent performance and conditional 10-fold out-of-fold performance.

In the adenocarcinoma-restricted sensitivity analysis (52 category-4 adenocarcinoma cases versus 139 non-malignant controls; *n* = 191), Model 1 had an apparent AUC of 0.9322 (95% CI, 0.8914–0.9642), a conditional 10-fold out-of-fold AUC of 0.8925 (95% CI, 0.8403–0.9375), and a bootstrap-corrected AUC of 0.9020. Thus, discrimination was not materially attenuated when the analysis was restricted to category-4 adenocarcinoma records. Detailed model coefficients and performance metrics are presented in Table 5, and Figure 2 illustrates the apparent and 10-fold out-of-fold discriminative performance of the final Model 1.

## 4. Discussion

This study evaluated routine inflammation- and nutrition-related measures as discriminators of histopathologically confirmed gastric malignancy within an endoscopy-selected case–control cohort. Several markers showed group-level associations, and the primary multivariable model retained relatively high discrimination under conditional internal validation. The lower estimates from the selection-inclusive nested audit demonstrate that uncertainty from variable selection is material and should be considered when interpreting model performance.

Higher NLR, SII, and SIRI values in the malignancy group are compatible with previously described associations between systemic inflammation and cancer biology. However, the present retrospective comparisons do not identify tumor-microenvironment mechanisms or distinguish tumor-related inflammation from bleeding, infection, medication effects, or other contributors.

Lower HDL, albumin, and HALP values are compatible with inflammatory and nutritional disturbances in established disease. These observations do not establish their cause or specificity for gastric malignancy. Unmeasured treatment exposures and acute clinical conditions remain alternative explanations, particularly for HDL and coagulation measurements.

CNIS and IINI showed univariable associations with malignancy. The CNIS-based alternative specification did not show a detectable discrimination advantage over Model 1. Because the models replaced rather than simply added composite terms, this comparison does not directly test the incremental contribution of CNIS to an otherwise identical model. IINI was not entered into a corresponding primary multivariable comparison.

Chronic inflammation, including Helicobacter pylori–associated mucosal injury, has established biological links with gastric carcinogenesis [16,17]. In this cohort, NLR, SII, and SIRI showed group-level discrimination consistent with an inflammation-related pattern. Much of the cited inflammatory-index literature concerns prognosis rather than diagnosis [6,7,18]; it cannot establish transportable diagnostic thresholds or clinical utility for this endoscopy-selected sample.

Platelets and tumor-associated macrophages have recognized roles in cancer biology [19], providing a rationale for examining multicomponent inflammatory indices. Nevertheless, several indices share laboratory components, and their effects can be correlated. The bootstrap selection instability observed here argues against interpreting the retained variables as a uniquely stable biological signature or assuming that multicomponent indices necessarily outperform individual measurements.

Variables related to nutritional status and lipid metabolism also differed between the study groups. Lower albumin, total protein, and HALP values in the malignancy group may reflect systemic catabolic burden and reduced nutritional reserves. HALP integrates hemoglobin, albumin, lymphocyte, and platelet components and showed group-level discrimination in this cohort; however, its retained multivariable coefficient was not statistically significant, and the index should not be interpreted as an independently validated diagnostic marker [20].

The CONUT score was higher in the malignant group, but the comparison using its conventional nutritional-status categories was not statistically significant (*p* = 0.075). Separately, the continuous CONUT term was not statistically significant after multivariable adjustment (*p* = 0.348). These are distinct analyses; neither establishes absence of nutritional differences or equivalence between groups.

Lower HDL cholesterol was associated with malignancy in both univariable and multivariable analyses. However, statin exposure was not available as a systematically recorded variable in the analytical database; therefore, the HDL association may be affected by residual confounding and should not be interpreted as tumor-specific or causal.

Higher INR values showed discrimination and a statistically significant multivariable association. The study-specific INR cut-off of 1.01 lies within commonly used reference ranges and represents only an internally derived exploratory threshold, not a pathological or clinical decision limit. Because anticoagulant exposure was not systematically available in the analytical database, residual medication-related confounding cannot be excluded; mechanistic interpretations involving tumor-associated coagulation should therefore remain cautious [21].

The multivariable findings indicate that discrimination in this cohort was distributed across inflammatory, hematological, metabolic, and demographic information. They should not be interpreted as causal effects or as a clinically deployable risk score. Propensity score matching was not used because malignancy was the diagnostic target rather than an exposure assignment; matching would have altered the observed clinical case mix and reduced the available event count [12].

Higher RDW showed a statistically significant multivariable association with malignancy in the present model. RDW reflects anisocytosis and has been associated with inflammation, oxidative stress, impaired iron metabolism, and malnutrition across solid tumors. This finding supports RDW as one component of the observed multivariable discrimination but does not, by itself, establish clinical utility [22].

Lower MPV showed a statistically significant multivariable association with malignancy, an observation relevant to ongoing debate regarding platelet indices in cancer. Reduced MPV may reflect altered platelet production, consumption, or inflammatory turnover; however, these mechanisms were not measured in this study. MPV should therefore be interpreted as one component of the multivariable pattern rather than as an isolated diagnostic marker [23].

Shared components and correlations may partly explain why the CNIS-based alternative and Model 1 showed similar apparent discrimination. These observations do not demonstrate incremental clinical value for CNIS or IINI. Independent validation and assessment of calibration and decision consequences would be needed before considering clinical application [24].

A strength of this study is the use of non-malignant endoscopic controls rather than healthy volunteers, with the same endoscopic and histopathological reference standard applied to all participants. Premalignant lesions were correctly retained within the non-malignant outcome, and analyses excluding these lesions and restricting the malignant group to adenocarcinoma produced similar discrimination estimates. These analyses support the internal robustness of the group-level comparison but do not establish population screening performance or clinical utility.

In this study, we comprehensively evaluated the diagnostic value of systemic inflammation- and nutrition-based indices derived from routine laboratory parameters in differentiating gastric malignancy. Our findings suggest that several inflammatory and hematological markers are associated with gastric malignancy and that the proposed multivariable model demonstrated high apparent discriminative performance, which remained robust after internal validation.

The observed results further support the central role of systemic inflammation in gastric cancer biology. In particular, significantly elevated levels of the neutrophil-to-lymphocyte ratio (NLR), systemic immune-inflammation index (SII), and systemic inflammation response index (SIRI) in the malignant group are consistent with an enhanced inflammatory milieu and immune dysregulation within the tumor microenvironment. Given the tumor-promoting effects of neutrophils and the critical role of lymphocytes in antitumor immunity, disruption of this balance likely contributes to a tumor-favorable environment.

The markedly reduced high-density lipoprotein (HDL) levels observed in malignant patients also highlight the interplay between lipid metabolism and tumor biology. Low HDL levels have been associated with chronic inflammation and increased oxidative stress, both of which may facilitate metabolic reprogramming in tumor cells. Similarly, decreased albumin levels and lower Hemoglobin–Albumin–Lymphocyte–Platelet (HALP) index values reflect the combined impact of malnutrition and systemic inflammation, both of which are well-established features of cancer-associated metabolic derangement.

When composite indices were examined, both the Composite Nutrition–Inflammation Score (CNIS) and the Inflammation–Immune–Nutrition Index (IINI) showed significant associations with malignancy in univariable analyses. However, these indices did not provide additional predictive value in multivariable models. This finding suggests that core predictors with stronger and more independent biological signals may dominate model performance, and that increasing model complexity through composite indices does not necessarily translate into improved discrimination. From a clinical perspective, this supports the use of more parsimonious and interpretable models.

The relationship between systemic inflammation and gastric carcinogenesis has been well established. Chronic inflammation, particularly in the context of Helicobacter pylori–associated mucosal injury, promotes tumor development through multiple mechanisms, including cytokine activation, oxidative stress, epithelial–mesenchymal transition, and immune evasion [16,17]. However, the clinical relevance lies in determining the extent to which these biological processes can be captured using routine laboratory parameters. Our findings indicate that indices reflecting neutrophil predominance and relative lymphopenia provide superior discriminative performance. The highest AUC observed for NLR in our study is consistent with previous reports evaluating the diagnostic value of inflammatory markers in gastric cancer. Similarly, the strong performance of SII and SIRI suggests that, beyond neutrophil–lymphocyte balance, platelet and monocyte components also contribute meaningfully to the biological landscape of gastric malignancy [18].

Platelets are known to facilitate tumor cell survival in circulation, promote endothelial adhesion, and contribute to the formation of metastatic niches, whereas the monocyte–tumor-associated macrophage axis plays a critical role in angiogenesis, stromal remodeling, and immune suppression [19]. Accordingly, multi-component indices such as SII, SIRI, and PIV are theoretically expected to outperform single parameters. In our study, all these indices were significantly elevated in the malignant group. However, in the multivariable setting, individual parameters with stronger independent effects were preferentially retained. This observation likely reflects partial collinearity among inflammation-related indices and overlapping biological information. In other words, although inflammatory indices provide robust discrimination at the univariable level, multivariable modeling tends to retain the most stable and non-redundant signals representing the underlying biological axis.

One of the notable findings of this study is the diagnostic contribution of variables related to nutritional status and lipid metabolism. The significantly lower levels of albumin, total protein, and particularly the HALP index in malignant patients reflect the systemic catabolic burden and depletion of nutritional reserves associated with gastric cancer. As a composite index integrating hemoglobin, albumin, lymphocyte, and platelet components, HALP simultaneously captures both inflammatory and nutritional dimensions, making it a practical and biologically integrative marker. Although HALP has been widely investigated in gastrointestinal malignancies, our findings support its relevance in the diagnostic discrimination setting by demonstrating its ability to differentiate gastric malignancy cases from non-malignant endoscopic controls [20].

Similarly, although the CONUT score was higher in the malignant group, its lack of independent contribution in categorical analyses suggests that nutritional impairment, while relevant, may represent a less specific signal compared to inflammation-based markers in the context of gastric malignancy.

The relationship between lipid metabolism and tumor biology has attracted increasing attention in recent years. In particular, low high-density lipoprotein (HDL) cholesterol levels have been associated with chronic inflammation, increased oxidative stress, and tumor-related metabolic reprogramming [25]. In our study, low HDL not only demonstrated a high AUC in univariable ROC analysis but also remained an independent predictor in the multivariable model. This finding is clinically relevant, as HDL is often regarded as a secondary biochemical parameter in oncological studies; however, our results suggest that it may play a more central role in the biological landscape of gastric malignancy.

Similarly, the strong performance of international normalized ratio (INR) may reflect the complex interplay between systemic inflammation, hepatic synthetic function, nutritional status, and tumor-associated coagulation activation. The association between cancer and hypercoagulability is well established, and alterations in the coagulation system may reflect systemic tumor-related biological activity [21].

Multivariable modeling represents one of the most clinically relevant outputs of our study. Following LASSO-based variable selection, ESR, RDW, MPV, HDL, INR, and male sex emerged as independent predictors of gastric malignancy. These findings indicate that gastric cancer risk is better captured through a multidimensional framework incorporating systemic inflammation, erythrocyte heterogeneity, platelet activity, lipid metabolism, and demographic factors, rather than relying solely on conventional tumor markers.

Notably, RDW remained an independent predictor, highlighting its potential clinical utility. Although RDW is a simple hematological parameter reflecting anisocytosis, it has been closely associated with chronic inflammation, oxidative stress, impaired iron metabolism, and malnutrition. Recent studies have increasingly reported the clinical relevance of RDW across various solid tumors, and our findings further support its relevance in the diagnostic context of gastric malignancy [22].

The observation that lower MPV levels independently predicted malignancy adds to an area of ongoing debate in the literature. While some studies have associated elevated MPV with inflammatory activity, others suggest that reduced MPV may reflect increased platelet consumption and heightened inflammatory turnover in oncological settings [23]. In our cohort, the association between lower MPV and malignancy may indicate an altered platelet production–consumption balance in gastric cancer. Therefore, MPV may be more informative when interpreted in conjunction with other inflammatory markers rather than as an isolated parameter.

Another strength of this study lies in the development of composite inflammation–nutrition indices. Both CNIS and IINI were significantly elevated in malignant patients and demonstrated meaningful discriminative performance in univariable analyses. However, the lack of incremental benefit when CNIS was incorporated into multivariable models suggests two potential interpretations. First, although biologically meaningful, composite indices may share substantial variance with individual predictors already included in the model. Second, while such indices provide a simplified representation of complex biological processes, their added value may be limited in the presence of strong individual predictors. This finding reflects the well-recognized balance between theoretical integrative modeling and statistical parsimony in biomarker research [24].

This study has several notable strengths. The use of a clinically relevant control group—comprising patients in whom malignancy was excluded through endoscopy and histopathology rather than healthy individuals—enhances the real-world applicability of the findings. Importantly, the control group should be interpreted as a non-malignant endoscopic control group rather than a healthy or purely benign population. This distinction is clinically relevant because the study population reflects patients who underwent endoscopic evaluation in routine practice. Premalignant lesions were not classified as malignant outcomes because they do not represent invasive cancer, and sensitivity analysis excluding these lesions yielded materially similar results. Therefore, the inclusion of premalignant lesions within the non-malignant control group did not appear to drive the observed diagnostic associations. Furthermore, the combined use of individual biomarkers, composite indices, and advanced statistical modeling techniques strengthens the robustness and clinical relevance of the results.

### Limitations

The retrospective, single-center, endoscopy-selected case–control design limits transportability of discrimination, calibration, PPV, NPV, and clinical utility. Consecutive recruitment and the distribution of endoscopy indications were not established. Exclusion of 16 patients with incomplete laboratory data may introduce selection bias; their variable-level missingness and group distribution were not available. Detailed lesion-level histology and confirmation of four database EGC labels were unavailable. Forty-nine of 72 malignant cases (68.1%) had TNM III–IV disease, precluding claims about early or asymptomatic detection. Although the three-day pretreatment sampling window and clinical assessment procedures were clarified, structured bleeding, medication, and localized-infection data were insufficient for reliable adjustment or exclusion-based sensitivity analysis. Separate localized-infection exclusion counts and their overlap with systemic infections were not established. Other assay reference intervals were unavailable. With 72 events and 12 primary-model parameters, overfitting remains important; the historical selection procedure was not fully reproducible. Fixed-set validation omits selection uncertainty, and the separately specified nested audit showed lower discrimination, poor calibration, and unstable selection. Its inclusion of Helicobacter pylori status of unconfirmed ascertainment timing further limits interpretation as a pre-endoscopic tool. Prospective external validation in a consecutively recruited target population is required [14,26].

## 5. Conclusions

In this endoscopy-selected retrospective cohort, routine inflammation- and nutrition-related laboratory measures showed discriminatory ability between histopathologically confirmed gastric malignancy and non-malignant endoscopic findings. These associations may partly reflect the burden of clinically established disease and should not be interpreted as evidence of early-cancer screening performance.

The primary multivariable model showed high apparent discrimination and retained relatively high performance under conditional internal validation; a fully nested selection-inclusive audit produced more conservative estimates. Similar results after exclusion of premalignant controls and restriction to adenocarcinoma supported the primary group-level interpretation. The model is exploratory, is not a replacement for endoscopy and histopathology, and requires prospective external validation before any clinical use.

## Figures and Tables

**Table 1:** Baseline demographic and clinical characteristics of gastric malignancy cases and non-malignant endoscopic controls.

Variable	Total (*n* = 211)	Non-Malignant Controls (*n* = 139)	Gastric Malignancy (*n* = 72)	Effect Size (d Equivalent)	*p*
Age, _mean (SD)_	60.67 (13.18)	57.44 (12.80)	66.92 (11.63)	0.8	<0.001 †
Sex, *_n_*_(%)_				0.6	<0.001 ^b^
Female	91 (43.1%)	74 (53.2%)	17 (23.6%)		
Male	120 (56.9%)	65 (46.8%)	55 (76.4%)		
Helicobacter pylori Status, *_n_*_(%)_					0.999 ^b^
Absent	120 (56.9%)	79 (56.8%)	41 (56.9%)		
Present	91 (43.1%)	60 (43.2%)	31 (43.1%)		
Comorbidity Burden, *_n_*_(%)_					0.335 ^b^
None	39 (18.5%)	29 (20.9%)	10 (13.9%)		
One Disease	58 (27.5%)	41 (29.5%)	17 (23.6%)		
2–3 Diseases	69 (32.7%)	41 (29.5%)	28 (38.9%)		
≥4 Diseases	45 (21.3%)	28 (20.1%)	17 (23.6%)		
Comorbidity Presence, *_n_*_(%)_					0.263 ^b^
No	39 (18.5%)	29 (20.9%)	10 (13.9%)		
Yes	172 (81.5%)	110 (79.1%)	62 (86.1%)		
History of Extra Malignancy, *_n_*_(%)_					0.279 ^c^
No	202 (95.7%)	135 (97.1%)	67 (93.1%)		
Yes	9 (4.3%)	4 (2.9%)	5 (6.9%)		
Number of Comorbidities, _median_ _(Q1/Q3)_	2.00 (1.00/3.00)	1.00 (1.00/3.00)	2.00 (1.00/3.00)		0.236 ^a^

^a^ Mann–Whitney U test (Monte Carlo), † Independent Samples *T* Test (Bootstrap), ^b^ Chi-square test, ^c^ Fisher Freeman Halton test; Post Hoc Test: Benjamini–Hochberg correction, SD: Standard Deviation, Med: Median, Q1: 1st Quartile, Q3: 3rd Quartile. Effect sizes were initially calculated according to variable type: Cohen’s *d* for continuous variables, rank-biserial correlation for Mann–Whitney U comparisons, phi coefficient for 2 × 2 categorical variables, and Cramér’s V for larger contingency tables. To facilitate consistency and comparability across variables, non-*d* effect sizes were subsequently transformed into an equivalent Cohen’s d metric using standard conversion formulas. Conversions derived from categorical variables were interpreted as approximate estimates. Non-malignant endoscopic controls comprised patients with benign or premalignant histopathological findings and no evidence of invasive gastric malignancy.

**Table 3:** Comparison of inflammatory, nutritional, and composite indices between gastric malignancy cases and non-malignant endoscopic controls, with standardized effect sizes.

Variable	Total (*n* = 211)	Non-Malignant Controls (*n* = 139)	Gastric Malignancy (*n* = 72)	Effect Size (d Equivalent)	*p*
PIV, median (Q1/Q3)	294.31 (160.70/564.09)	213.50 (142.62/410.84)	501 (261.18/1137.05)	1.1	<0.001 ^a^
CAR, median (Q1/Q3)	0.18 (0.09/0.46)	0.15 (0.07/0.34)	0.37 (0.18/0.84)	0.9	<0.001 ^a^
CONUT Score, median (Q1/Q3)	2 (0.50/4)	1 (0/3)	3 (1/5)	0.6	<0.001 ^a^
HALP Index, median (Q1/Q3)	34.55 (19.13/51.66)	38.88 (23.63/55.85)	22.46 (13.66/36.23)	0.9	<0.001 ^a^
Nutrition Z score, median (Q1/Q3)	−0.14 (−0.56/0.51)	−0.32 (−0.67/0.34)	0.22 (−0.25/0.86)	0.8	<0.001 ^a^
CNIS, median (Q1/Q3)	−1.04 (−2.52/1.54)	−1.54 (−2.94/0.30)	0.53 (−1.25/3.20)	1.0	<0.001 ^a^
IINI, median (Q1/Q3)	−0.22 (−1.54/1.10)	−0.94 (−1.86/0.56)	0.48 (−0.51/2.12)	0.9	<0.001 ^a^
SII, median (Q1/Q3)	504.84 (321/891.15)	420.4 (281/645.6)	814.1 (545.1/1424.5)	1.2	<0.001 ^a^
SIRI, median (Q1/Q3)	1.11 (0.66/2.08)	0.88 (0.6/1.4)	1.98 (1.25/3.4)	1.4	<0.001 ^a^
ESR, mm/h, median (Q1/Q3)	11 (5/22)	8 (4.50/17)	20 (10/34.25)	1.0	<0.001 ^a^
CEA, ng/mL, median (Q1/Q3)	1.81 (1.21/2.5)	1.61 (1.15/2.12)	2.51 (1.56/3.77)	0.9	<0.001 ^a^
NLR, median (Q1/Q3)	2 (1.12/3.47)	1.54 (1/2.3)	3.46 (2.31/5.04)	1.4	<0.001 ^a^
PLR, median (Q1/Q3)	136.9 (100.8/202)	125.9 (93.4/171.9)	166.8 (126.5/262.2)	0.7	<0.001 ^a^
CNIS Risk Group, *n* (%)				0.6	<0.001^b^
Low	53 (25.1%)	44 (31.7%)	9 (12.5%)		0.004
Intermediate	105 (49.8%)	72 (51.8%)	33 (45.8%)		0.469
High	53 (25.1%)	23 (16.5%)	30 (41.7%)		<0.001
CONUT Group *n* (%)					0.075 ^c^
Mild	70 (33.2%)	42 (30.2%)	28 (38.9%)		
Moderate	38 (18.0%)	21 (15.1%)	17 (23.6%)		
Normal	99 (46.9%)	74 (53.2%)	25 (34.7%)		
Severe	4 (1.9%)	2 (1.4%)	2 (2.8%)		
PIV Group, *n* (%)				0.7	<0.001 ^b^
High	105 (49.8%)	53 (38.1%)	52 (72.2%)		
Low	106 (50.2%)	86 (61.9%)	20 (27.8%)		
CAR Group, *n* (%)				0.6	<0.001 ^b^
High	105 (49.8%)	54 (38.8%)	51 (70.8%)		
Low	106 (50.2%)	85 (61.2%)	21 (29.2%)		
HALP Group, *n* (%)				0.6	<0.001 ^b^
High	105 (49.8%)	83 (59.7%)	22 (30.6%)		
Low	106 (50.2%)	56 (40.3%)	50 (69.4%)		

^a^ Mann–Whitney U test (Monte Carlo), ^b^ Chi-square test, ^c^ Fisher Freeman Halton test; Post Hoc Test: Benjamini–Hochberg correction, Q1: 1st Quartile, Q3: 3rd Quartile. Effect sizes were initially computed according to variable type: Cohen’s d for continuous variables, rank-biserial correlation for Mann–Whitney U comparisons, phi coefficient for 2 × 2 categorical variables, and Cramér’s V for larger contingency tables. For consistency and comparability across variables, these effect sizes were subsequently transformed to an equivalent Cohen’s d metric using standard conversion formulas. Con-versions derived from categorical variables were interpreted as approximate estimates.

**Table 5:** LASSO-selected multivariable logistic regression models for discriminating gastric malignancy cases from non-malignant endoscopic controls.

Variables	β (SE)	OR (95% CI)	*p*
MODEL 1			
Age, per 1-SD increase	0.401 (0.246)	1.493 (0.922–2.417)	0.103
ESR, per 1-SD increase	0.738 (0.266)	2.091 (1.242–3.521)	0.006
Hematocrit, per 1-SD increase	−0.364 (0.284)	0.695 (0.398–1.214)	0.201
RDW, per 1-SD increase	0.610 (0.268)	1.841 (1.088–3.114)	0.023
MPV, per 1-SD increase	−0.713 (0.258)	0.490 (0.296–0.814)	0.006
HDL cholesterol, per 1-SD increase	−1.257 (0.389)	0.284 (0.133–0.609)	0.001
ALT, per 1-SD increase	−0.444 (0.264)	0.641 (0.382–1.076)	0.093
ALP, per 1-SD increase	0.367 (0.290)	1.443 (0.817–2.550)	0.206
INR, per 1-SD increase	1.178 (0.517)	3.248 (1.179–8.951)	0.023
Sex: male vs. female (reference)	1.537 (0.511)	4.652 (1.708–12.672)	0.003
CONUT score, per 1-SD increase	−0.263 (0.280)	0.768 (0.444–1.331)	0.348
HALP: high vs. low (reference)	0.315 (0.568)	1.371 (0.450–4.174)	0.579
Model 1 performance	Apparent AUC = 0.9276 (95% CI, 0.8896–0.9589); AIC = 161.948; Youden threshold = 0.4698; sensitivity = 81.9%; specificity = 89.2%; PPV = 79.7%; NPV = 90.5%; accuracy = 86.7%; Brier = 0.102. Fixed-set validation: 10-fold OOF AUC = 0.8897 (95% CI, 0.8406–0.9307); bootstrap-corrected AUC = 0.9009; OOF calibration intercept = −0.128; slope = 0.712.		
MODEL 2			
Age, per 1-SD increase	0.411 (0.249)	1.508 (0.927–2.455)	0.098
ESR, per 1-SD increase	0.768 (0.269)	2.155 (1.272–3.651)	0.004
Hematocrit, per 1-SD increase	−0.357 (0.270)	0.700 (0.412–1.188)	0.186
RDW, per 1-SD increase	0.612 (0.263)	1.844 (1.102–3.087)	0.020
MPV, per 1-SD increase	−0.725 (0.257)	0.484 (0.293–0.801)	0.005
HDL cholesterol, per 1-SD increase	−1.273 (0.382)	0.280 (0.132–0.593)	<0.001
ALT, per 1-SD increase	−0.389 (0.246)	0.678 (0.418–1.098)	0.114
ALP, per 1-SD increase	0.347 (0.289)	1.414 (0.803–2.490)	0.230
INR, per 1-SD increase	1.126 (0.497)	3.082 (1.164–8.161)	0.023
Sex: male vs. female (reference)	1.542 (0.510)	4.673 (1.721–12.688)	0.002
CNIS: intermediate vs. high (reference)	0.495 (0.554)	1.640 (0.554–4.855)	0.371
CNIS: low vs. high (reference)	0.976 (0.798)	2.654 (0.555–12.681)	0.221
CNIS: low vs. intermediate (post-estimation contrast)	0.481 (0.644)	1.618 (0.458–5.723)	0.455
Model 2 performance	Apparent AUC = 0.9272 (95% CI, 0.8896–0.9580); AIC = 161.922; Youden threshold = 0.5089; sensitivity = 77.8%; specificity = 92.1%; PPV = 83.6%; NPV = 88.9%; accuracy = 87.2%; Brier = 0.103. Fixed-set validation: 10-fold OOF AUC = 0.8914 (95% CI, 0.8446–0.9322); bootstrap-corrected AUC = 0.8996; OOF calibration intercept = −0.124; slope = 0.729.		
Paired bootstrap comparison, Model 1 minus Model 2: ΔAUC = 0.0004 (95% CI, −0.0053 to 0.0063), *p* = 0.888.			

Refitted multivariable logistic regression using the originally reported LASSO-selected predictor sets. Continuous-variable coefficients and odds ratios are reported per one-standard-deviation increase on the original variable scale; negative coefficients indicate lower odds with increasing values. Categorical reference groups are shown explicitly; the low-versus-intermediate CNIS row is a post-estimation contrast rather than an additional model parameter. LASSO-selected variables with *p* ≥ 0.05 are retained model terms but are not described as statistically significant independent associations. Fixed-set internal validation conditions on the selected predictor set and does not include selection uncertainty. PPV and NPV reflect the case–control sample malignancy proportion. Full model intercepts, scaling constants and categorical coding are supplied in Supplementary Section S3. Post-selection Wald inference is descriptive.

## Data Availability

The data that support the findings of this study are available on request from the corresponding author.

## Supplementary Materials

The following supporting information can be downloaded at https://www.mdpi.com/article/10.3390/life16091547/s1: Section S1: Participant flow and missing data; Figure S1: Participant disposition; Table S1: Candidate-variable completeness; Section S2: Validation specification; Section S3: Primary model equations and coding; Table S2: Model 1; Table S3: Model 2; Section S4: Precision-sensitive univariable cut-offs; Table S4: Unrounded thresholds and classification counts; Section S5: TRIPOD + AI reporting checklist.

## Abbreviations

The following abbreviations are used in this manuscript:

NLR	Neutrophil-to-lymphocyte ratio
PLR	Platelet-to-Lymphocyte Ratio
SII	Systemic immune-inflammation index
SIRI	Systemic inflammation response index
CONUT	Controlling nutritional status score
HALP	Hemoglobin–Albumin–Lymphocyte–Platelet index
PNI	Prognostic Nutritional Index
PIV	Pan-immune-inflammation value
CAR	C-reactive protein-to-albumin ratio
CNIS	Composite Nutrition–Inflammation Score
IINI	Inflammation–Nutrition Index
ROC	Receiver operating characteristic
LASSO	Least absolute shrinkage and selection operator
AUC	Area under the curve
CRP	C-reactive protein
ESR	Erythrocyte sedimentation rate
CEA	Carcinoembryonic Antigen
ALT	Alanine Transaminase
AST	Aspartate Transaminase
GGT	Gamma-Glutamyl Transferase
ALP	Alkaline Phosphatase
INR	International normalized ratio
HDL	High-density lipoprotein
LDL	Low-density lipoprotein
RDW	Red cell distribution width
MCV	Mean corpuscular volume
MCH	Mean corpuscular hemoglobin
MPV	Mean platelet volume
