# Supplementary material for: Discriminative Performance of Routine Inflammation- and Nutrition-Based Indices for Histopathologically Confirmed Gastric Malignancy: A Retrospective Case–Control Study
Source: Life (Basel). 2026 Sep 16;16(9):1547. doi: 10.3390/life16091547 (PMC13608704; doi:10.3390/life16091547)
Supplement: Supplementary file 1 [file life-16-01547-s001.zip › life-4519285-supplementary.pdf]

Supplementary Material

Discriminative Performance of Routine Inflammation- and Nutrition-Based Indices for Histopathologically Confirmed Gastric Malignancy: A Retrospective Case–Control Study

Reporting clarifications, reproducibility details, and checklist. This supplement accompanies the revised manuscript; unavailable source information is explicitly identified rather than inferred.

S1. Participant flow and missing data

Supplementary Figure S1. Participant disposition

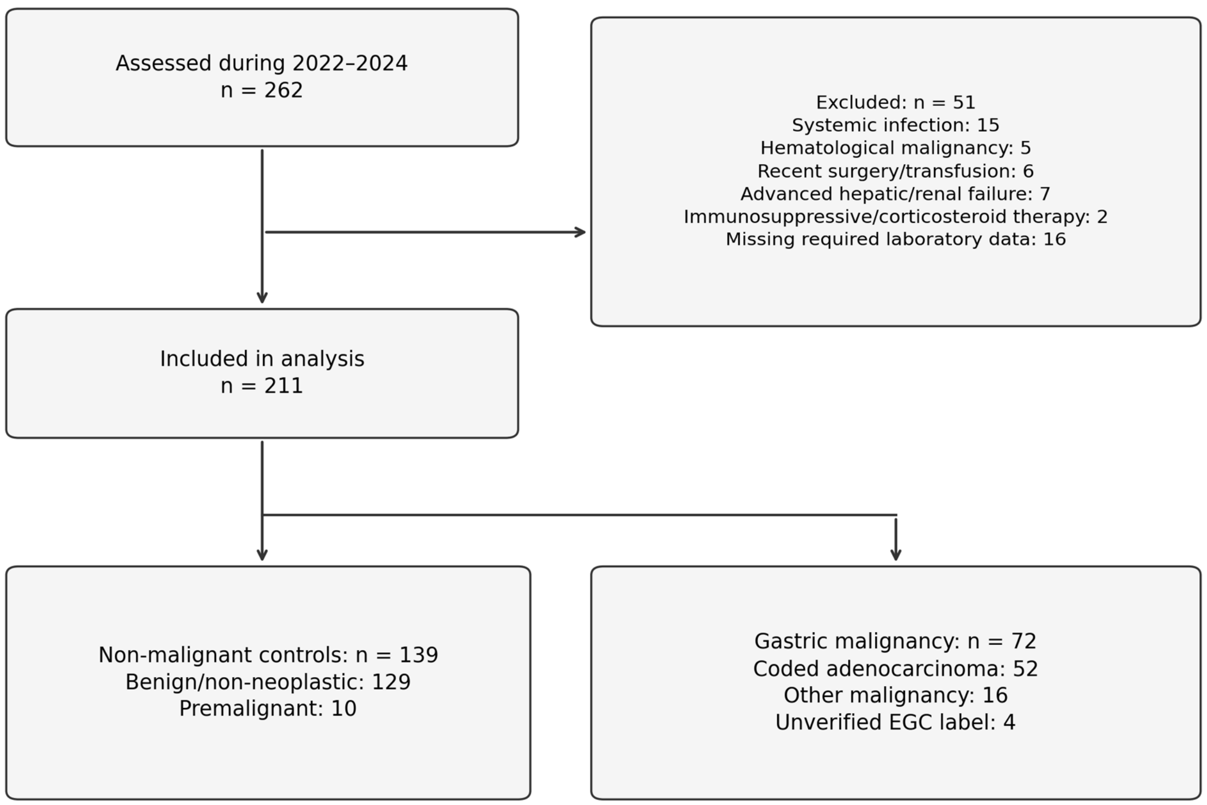

Counts reproduce the investigators' supplied exclusion accounting: 15+5+6+7+2+16=51; 262−51=211. A separate localized-infection count and its overlap with systemic infection were unavailable; no additional exclusion count is inferred. Consecutive recruitment and a standardized indication distribution were not established. EGC labels do not establish verified early-cancer diagnoses; TNM stage alone cannot resolve them.

Supplementary Table S1. Candidate-variable completeness

Variable	Controls missing/n	Cases missing/n	Total missing/n

Variable	Controls missing/n	Cases missing/n	Total missing/n
Age	0/139 (0%)	0/72 (0%)	0/211 (0%)
Sex	0/139 (0%)	0/72 (0%)	0/211 (0%)
Helicobacter pylori Status	0/139 (0%)	0/72 (0%)	0/211 (0%)
Number of Comorbidities	0/139 (0%)	0/72 (0%)	0/211 (0%)
History of Extra Malignancy	0/139 (0%)	0/72 (0%)	0/211 (0%)
PIV	0/139 (0%)	0/72 (0%)	0/211 (0%)
CAR	0/139 (0%)	0/72 (0%)	0/211 (0%)
CONUT Score	0/139 (0%)	0/72 (0%)	0/211 (0%)
HALP Index	0/139 (0%)	0/72 (0%)	0/211 (0%)
SII	0/139 (0%)	0/72 (0%)	0/211 (0%)
SIRI	0/139 (0%)	0/72 (0%)	0/211 (0%)
ESR	0/139 (0%)	0/72 (0%)	0/211 (0%)
CEA	0/139 (0%)	0/72 (0%)	0/211 (0%)
NLR	0/139 (0%)	0/72 (0%)	0/211 (0%)
PLR	0/139 (0%)	0/72 (0%)	0/211 (0%)
WBC	0/139 (0%)	0/72 (0%)	0/211 (0%)
Hemoglobin	0/139 (0%)	0/72 (0%)	0/211 (0%)
Hematocrit	0/139 (0%)	0/72 (0%)	0/211 (0%)
Platelet Count	0/139 (0%)	0/72 (0%)	0/211 (0%)
MCV	0/139 (0%)	0/72 (0%)	0/211 (0%)
MCH	0/139 (0%)	0/72 (0%)	0/211 (0%)
MCHC	0/139 (0%)	0/72 (0%)	0/211 (0%)
RDW	0/139 (0%)	0/72 (0%)	0/211 (0%)
RDW SD	0/139 (0%)	0/72 (0%)	0/211 (0%)
MPV	0/139 (0%)	0/72 (0%)	0/211 (0%)
Neutrophil Count	0/139 (0%)	0/72 (0%)	0/211 (0%)
Lymphocyte Count	0/139 (0%)	0/72 (0%)	0/211 (0%)
Monocyte Count	0/139 (0%)	0/72 (0%)	0/211 (0%)

Variable	Controls missing/n	Cases missing/n	Total missing/n
Eosinophil Count	0/139 (0%)	0/72 (0%)	0/211 (0%)
Basophil Count	0/139 (0%)	0/72 (0%)	0/211 (0%)
HDL Cholesterol	0/139 (0%)	0/72 (0%)	0/211 (0%)
LDL Cholesterol	0/139 (0%)	0/72 (0%)	0/211 (0%)
Triglycerides	0/139 (0%)	0/72 (0%)	0/211 (0%)
Total Cholesterol	0/139 (0%)	0/72 (0%)	0/211 (0%)
CRP	0/139 (0%)	0/72 (0%)	0/211 (0%)
ALT	0/139 (0%)	0/72 (0%)	0/211 (0%)
AST	0/139 (0%)	0/72 (0%)	0/211 (0%)
GGT	0/139 (0%)	0/72 (0%)	0/211 (0%)
ALP	0/139 (0%)	0/72 (0%)	0/211 (0%)
INR	0/139 (0%)	0/72 (0%)	0/211 (0%)
Urea	0/139 (0%)	0/72 (0%)	0/211 (0%)
Creatinine	0/139 (0%)	0/72 (0%)	0/211 (0%)
Total Protein	0/139 (0%)	0/72 (0%)	0/211 (0%)
Albumin	0/139 (0%)	0/72 (0%)	0/211 (0%)
Calcium	0/139 (0%)	0/72 (0%)	0/211 (0%)

All 45 candidates are complete in the included sample. No imputation was used. Sixteen of 262 assessed patients (6.1%) were excluded for missing required laboratory data; the variable-level pattern and case/control status of those excluded patients were unavailable. This table therefore describes the included sample only, not pre-exclusion completeness or an absence of selection bias.

S2. Validation specification

The primary fixed-set analyses refitted scaling, category cut-points, and logistic coefficients within each of ten stratified training folds and 500 bootstrap samples, retaining the originally reported predictor set. These estimates are conditional on that set. The original LASSO code and tuning trace were unavailable.

The separate selection-inclusive audit used the 45 candidates in Table S1 on their recorded scales, without log transformation. All scaling was learned within training data. L1 logistic regression (liblinear) searched 12 C values equally spaced on a base-10 logarithmic scale from −2.5 to 0.8. Inner stratified five-fold cross-validation evaluated ROC AUC. The smallest C within one standard error of the best mean AUC was selected; nonzero terms (absolute coefficient >10^−8) were refitted without penalization. Preprocessing, tuning, selection and refitting were repeated within every outer ten-fold training set and bootstrap sample. The configured fallback for a zero-feature fit selected the candidate with the largest

absolute outcome correlation within training data; it was not based on held-out outcomes. The principal reproducibility seed was 20260902. This procedure is a new methodological sensitivity analysis, not recovery of the historical selection procedure.

Bootstrap optimism was the average difference between each bootstrap-developed model's AUC in its bootstrap sample and in the original sample; corrected AUC subtracts this average from apparent AUC. AUC confidence limits were percentile limits from 5,000 class-stratified bootstrap samples of the relevant predictions. Resampling fixed out-of-fold predictions does not capture every source of model-development variability. Model comparison used paired resampling. No oversampling, outcome weighting, or synthetic cases were used. The unknown ascertainment time of Helicobacter pylori status limits interpreting the audit as wholly pre-endoscopic.

Analysis	Apparent AUC	OOF AUC	Corrected AUC
Model 1; fixed set	0.9276	0.8897	0.9009
Model 2; fixed set	0.9272	0.8914	0.8996
Separate nested audit	0.9273	0.8730	0.8694
Adenocarcinoma restriction; Model 1	0.9322	0.8925	0.9020

OOF: out-of-fold. Corrected: bootstrap optimism-corrected. The fixed-set and selection-inclusive analyses answer different validation questions. The nested audit calibration slope of 0.406 and median 21 selected terms (IQR 17–26) indicate overfitting and instability. Stage-specific AUCs use subsets of full-cohort OOF predictions and are not validations of separately fitted stage models.

The audit fitted to the full cohort selected 12 terms: age, sex, ESR, hematocrit, MCV, RDW, MPV, lymphocyte count, basophil count, HDL cholesterol, ALP, and INR. This set differs from the primary Model 1 specification and is not proposed as a replacement clinical model.

S3. Primary model equations and coding

These specifications are for reproducibility, not clinical implementation. Let $z_j=(x_j-\text{mean}_j)/SD_j$ and $\eta=\text{intercept}+\Sigma\beta_j z_j+\text{categorical terms}$. The model output is $\exp(\eta)/(1+\exp(\eta))$. Continuous coefficients below refer to an increase on the original scale, matching Table 5; SD_j uses ddof=0. Outcome coding is 1=gastric malignancy, 0=non-malignant control. The case–control sample intercept is not a calibrated population-risk intercept.

Supplementary Table S2. Model 1

Term	Coefficient β	Full-cohort mean	Full-cohort SD
Intercept	-2.315660015	—	—
Age	0.400715178	60.672985782	13.149241057
ESR	0.737552039	16.697156398	16.461337483
Hematocrit	-0.363834826	37.163507109	6.421353966
RDW	0.610329771	15.978672986	3.563795057

Term	Coefficient β	Full-cohort mean	Full-cohort SD
MPV	-0.712535614	9.084834123	1.243087940
HDL Cholesterol	-1.257216387	52.985781991	29.481374741
ALT	-0.444165307	17.616113744	12.634096875
ALP	0.366998912	93.834123223	86.257060823
INR	1.178149007	1.034075829	0.276226217
CONUT Score	-0.262808538	2.454976303	2.428427880
Sex: male vs female	1.537397664	Binary 0/1	—
HALP: high vs low	0.315438081	Binary 0/1	—

Sex: male=1, female=0. HALP high=1 when HALP>34.5541237113; otherwise 0. The intercept and all coefficients are estimated together; the HALP split is distinct from the separate univariable Youden threshold in Table 4.

Supplementary Table S3. Model 2

Term	Coefficient β	Full-cohort mean	Full-cohort SD
Intercept	-2.649674818	—	—
Age	0.410878252	60.672985782	13.149241057
ESR	0.767587999	16.697156398	16.461337483
Hematocrit	-0.356783725	37.163507109	6.421353966
RDW	0.611949608	15.978672986	3.563795057
MPV	-0.725101842	9.084834123	1.243087940
HDL Cholesterol	-1.272638547	52.985781991	29.481374741
ALT	-0.389402586	17.616113744	12.634096875
ALP	0.346583177	93.834123223	86.257060823
INR	1.125560516	1.034075829	0.276226217
Sex: male vs female	1.541783594	Binary 0/1	—
CNIS: intermediate vs high	0.494797089	Binary 0/1	—
CNIS: low vs high	0.976073281	Binary 0/1	—

CNIS low: ≤-2.5185721184; intermediate: >-2.5185721184 and <1.54117376986; high: ≥1.54117376986 (reference). Low and intermediate indicators are 1 only for the corresponding category. Model 2 replaces CONUT and HALP with these two indicators; it does not add CNIS to the full Model 1.

CNIS component	Mean	Sample SD
PIV	736.098822743	2955.8786894
CAR	0.616091009931	1.50284101477
SII	1124.27872838	4151.45406652
NLR	3.63671221365	7.09892261003
CONUT	2.45497630332	2.43420298375
HALP	39.0929163189	29.9619393797
Albumin	3.82227488152	0.674802645071

For CNIS only, each component is standardized using the sample SD (ddof=1) above. CNIS=Z(PIV)+Z(CAR)+Z(SII)+Z(NLR)+Z(CONUT)−Z(HALP)−Z(albumin). These full-cohort constants must not be mistaken for externally validated clinical limits.

S4. Precision-sensitive univariable cut-offs

Supplementary Table S4. Unrounded thresholds and classification counts

Table 4 displays rounded cut-offs. Applying a rounded value literally can change classification for observations close to the boundary. The following unrounded thresholds reproduce the reported classification counts; all are internally derived exploratory results.

Marker	Calculation threshold (≥)	True positive /72	False positive /139
NLR	2.347826086956522	54	35
Nutrition Z-score	−0.2900241127638367	57	65
CNIS	−0.2937942819408158	49	43
IINI	−0.5630735703203037	56	58

For example, the inclusive INR threshold ≥1.01 gives 56/72 true positives and 42/139 false positives, matching sensitivity 77.78% and specificity 69.78%. Neither the numerical precision nor the direction makes any threshold a validated clinical decision rule.

S5. TRIPOD+AI reporting checklist

This item-by-item mapping uses concise paraphrased labels, not the full official checklist text. “Partial” means a reporting element remains unavailable or unverified; inclusion in this checklist does not establish complete compliance or low risk of bias. “Not applicable” is used only where the study did not undertake the specified activity. Official source: Collins et al., BMJ 2024;385:e078378; checklist: https://www.tripod-statement.org/wp-content/uploads/2019/12/TRIPODAI_checklist.pdf.

Item	Topic	Status	Location / explanation
1	Title	Reported	Title identifies outcome, discrimination and design.

Item	Topic	Status	Location / explanation
2	Abstract	Reported	Structured Abstract states sample, methods, results and limitations.
3a–c	Rationale and context	Partial	Introduction gives rationale and selected population; no formal healthcare-inequality assessment.
4	Objectives	Reported	Introduction: exploratory diagnostic discrimination, not screening.
5a–b	Data and dates	Partial	Methods: single-center retrospective records, 2022–2024. Representativeness/consecutive recruitment unestablished.
6a–c	Setting and eligibility	Partial	Methods and flow: eligibility and exclusions; pretreatment sampling. Full treatment exposure data unavailable.
7	Preparation	Reported	Methods and S1–S3: completeness, scaling, duplicate AISI/PIV, categorical definitions.
8a–c	Outcome assessment	Partial	Histopathological binary outcome stated. Detailed subtype resolution, assessor characteristics and blinding not documented.
9a–c	Predictor assessment	Partial	Candidates and definitions given. Blood timing known; H. pylori timing and assessor blinding not established.
10	Sample size	Partial	All 211 analytic records used; 72 events/12 primary parameters discussed. No documented prospective sample-size calculation.
11	Missingness	Partial	Included cohort complete; 16 exclusions. Excluded-patient variable/group patterns unavailable.
12a–c	Model development	Partial	Conditional and separately nested procedures specified; historical selection details unavailable.
12d	Clustering	Not applicable	Single-center analysis; no multi-cluster validation conducted.
12e	Performance	Reported	AUC with intervals, Brier, calibration, ROC figures and paired model comparison.
12f	Updating	Not applicable	No external model updating undertaken.
12g	Model output	Reported	S3 provides equations, intercepts, coefficients and coding.
13	Imbalance	Reported	Observed case/control counts retained; no synthetic sampling or weighting (S2).
14	Fairness	Not assessed	No fairness analysis conducted; clinical use is not proposed.
15	Thresholds	Reported	Youden thresholds explicitly exploratory; PPV/NPV sample-dependent; S4 precision notes.
16	Dataset differences	Not applicable	Internal resampling only; no separate external evaluation cohort.
17	Ethics	Reported	Statements: ethics approval, date/number and consent waiver.
18a–b	Support and conflicts	Reported	Statements: funding and conflicts declarations retained.

Item	Topic	Status	Location / explanation
18c–d	Protocol and registration	Not documented	Protocol access and registration status not established in supplied materials.
18e–f	Data and code	Partial	Data available on request per authors; model specification in S3. No public code repository documented; original selection code unavailable.
19	Public involvement	Not documented	Patient/public involvement status not established; no invented statement of involvement or absence.
20a–b	Participants	Partial	Figure S1 and Tables 1–3/S1; excluded-patient detail and some lesion subtypes unavailable.
20c	External comparison	Not applicable	No external evaluation dataset.
21	Analysis counts	Reported	211/72 primary, 201/72 excluding premalignancy, 191/52 adenocarcinoma restriction.
22	Full specification	Reported	Table 5 and S3 give primary model coefficients, intercepts and scaling; historical selection cannot be recovered.
23a	Performance results	Reported	Results and S2: apparent, conditional and selection-inclusive performance; exploratory stage subgroups.
23b	Between-cluster variation	Not applicable	No multi-cluster performance analysis.
24	Updating results	Not applicable	No model updating.
25	Interpretation	Partial	Discussion narrows conclusions; no fairness evidence is claimed.
26	Limitations	Reported	Selection, missingness, confounding, histology, overfitting and external validation gaps stated.
27a–c	Use and research	Reported for scope	Conclusion: not deployable, no replacement for endoscopy; prospective external validation required. No clinical workflow implemented.
